# Satisfaction and use of the didactic simulator for learning the nursing process: an observational study

**DOI:** 10.1186/s12912-024-01717-2

**Published:** 2024-01-12

**Authors:** Alberto Cruz-Barrientos, Eva Manuela Cotobal-Calvo, Ana María Sainz-Otero, José Manuel De-La-Fuente-Rodríguez, Daniel Román-Sánchez, Inés Carmona-Barrientos

**Affiliations:** 1 College Nursing Center Salus Infirmorum, Affiliated University of Cádiz, Cádiz, Spain; 2 University of Cádiz (UCA), Cádiz, Spain

**Keywords:** Satisfaction, Use, Nursing care, Learning, ICT

## Abstract

**Introduction:**

The implementation of Information and Communication Technology (ICT) in daily healthcare practice has become standardized. In relation to education within the nursing degree, ICTs make it possible to carry out practical immersion training from the” classroom or from any other place with an Internet connection, as evidenced by circumstances that have occurred in recent years, such as the pandemic caused by COVID-19.

**Objective:**

Design and assess a didactic simulation program for the training of the nursing process that promotes learning in the nursing care

**Methodology:**

The methodological approach is quantitative and it is a descriptive cross-sectional study. The sampling method used was non-probabilistic by convenience.

**Results:**

When observing the comparison of the averages of student satisfaction with respect to the didactic simulator, it is worth mentioning that all the items are above 2.80 on a score in which “0” is the minimum value and “5” the maximum value.

The results of the use of the computer tool by the students, we highlight as significant data that all the items present an average equal to or lower than 3.04 out of 7, where “1” corresponds to a lot of use and “7” to little use.

**Conclusion:**

The implication of ICT in the teaching–learning process among Nursing Degree students, it is considered optimal. when analyzing the answers obtained in the items that refer to a higher ease in the execution of development of Care plans, a fundamental work in Nursing performance.

## Introduction

The implementation of ICTs in daily healthcare practice has become standardized. As pointed out by different researchers [1, 2] their use ranges from the level of care [3, 4] to management [5, 6] research [7, 8] and, of course, training [9–11]. It should be noted that the use of these technologies reduces costs and errors and improves quality [12–15]. This phenomenon links nursing training and practice with information and communication technologies. These ICTs are part of the daily and working life of healthcare professionals. In relation to education within the nursing degree, ICTs make it possible to carry out practical immersion training from the classroom or from any other place with an Internet connection, as evidenced by circumstances that have occurred in recent years, such as the pandemic caused by COVID-19 [16–19].

The tool “Didactic Simulator for Learning the Nursing Process”  [20] (DISILNP) it facilitates the student’s assessment of the patient, using the Virginia Henderson model, as well as the use of the different standardized tests and questionnaires for nursing assessment. It uses the standardized nursing language of diagnoses, objectives and interventions. It also helps to perform a continuity of care simulation.

An example of this is the use of the Nursing Care Process (NCP), which allows humanistic, holistic, protocolized and scientifically evidenced patient care [21]. The NCP, in which students studying the nursing discipline are trained, requires theoretical knowledge and fundamental practical skills to subsequently practice the profession.

At present, there are not many digitalized tools that facilitate consultation and the use of standardized nursing language. Until 2009, there was no evidence related to an application that would help teachers and students to learn, develop, evaluate and better understand the NCP. It is only from 2012 that the “NNN Consult” [22] tool was put into operation with a methodology based on the North American Nursing Diagnosis Association (NANDA) taxonomy, together with the Nursing Outcome Classification (NOC) and Nursing Interventions Classification (NIC). As of 2015, a new version was implemented that included clinical cases and care plans. The “NNN Consult” is an electronic resource available on the internet, used by university students in their classes for learning the nursing process. This support allows the student to develop an individualized care plan, using the NANDA, NOC, and NIC Taxonomy. Currently, there is also another methodological tool like the one mentioned above, called “NANDA-I”. This electronic application is available on the website of the American Nursing Association, which developed the aforementioned Nursing Taxonomy on which we base our care plans. It is an electronic resource based on nursing diagnoses, objectives and interventions. In 2021, at the XI International Congress on University Teaching and Innovation, the “PAVCE Project: Virtual Learning Platform for Nursing Care” was presented, which allows a complete nursing care process to be carried out [23].

Learning through simulation, as pointed out by various studies, allows for learning in a safe environment where there is the possibility of trial and error without risks to a real patient [24–26].

Due to the rapid advancement of this type of education in the field of nursing, it becomes necessary to study the degree of student satisfaction with this type of education, as satisfaction can be a predictor of the effectiveness of simulation-based education [27–30].

The aim of this study was to develop a didactic simulation program for the training of the nursing process that promotes learning in the nursing care. To determine student satisfaction after using the didactic simulator in class by means of the EHRNS (Electronic Health Record Nurse Satisfaction) questionnaire. To measure the good use of the didactic simulator, the quality of the information and the quality of the interface by means of the PSSUQ (Post Study System Usability Questionnaire).

## Materials and methods

### Design and sample

The methodological approach is of a quantitative type and it is a transversal descriptive study.

Following the criteria of efficiency, the sampling method used was simple sampling, not probabilistic and for convenience.

The target population selected considered first-year and second-year Cadiz University Nursing Degree students, which consist of students of that are part of the Cadiz, Jerez, Algeciras campus as well as the ‘Salus Infirmorum’ University Nursing Centre, which is both a part of Cadiz University and is within the Cadiz campus. Out of a total of 660 stu-dents belonging to the 1st and 2nd year of the nursing degree, a final sample of 242 stu-dents was obtained. the criteria that defined the participation of the subjects were: stu-dents who wanted to participate in the study and gave their consent, students of 1st and 2nd year of Nursing Degree of the University of Cadiz and students who were in class at the time of the questionnaire. The exclusion criteria were students who did not bring a laptop or tablet, students who for some reason did not complete the questionnaire and students in the 3rd and 4th year of the Nursing Degree at the University of Cadiz.

### Data collection

Data collection was carried out from November 2016 to February 2017, through the resolution of a clinical case by the selected students, and the subsequent completion of the satisfaction questionnaire in paper format or through the Survey Monkey computer platform.

All participants were informed about the purposes of the study and signed an informed consent, therefore, this study complies with all ethical requirements established by the Declaration of Helsinki.

A questionnaire in paper format was used to complete the Test Battery or through the computer platform “Survey Monkey”. There were no conditions on face-to-face or on-line data collection. It was left to the student’s choice.

The study was conducted, firstly, by means of a self-drafted questionnaire that collected data on sociodemographic variables and variables related to the Nursing Degree (age, gender, marital status, province and city of origin, campus, course and system of access to the university).

Secondly, the “EHRNS” questionnaire, was used to measure nursing satisfaction after using the DISILNP. This questionnaire has a Cronbach’s alpha coefficient of 0.90. It consists of 21 questions with 6 subscales assessing structural quality, logistical quality of information, effects of quality on processes, effects of outcomes and program quality, unintended consequences/benefits and easy barriers/improvements. This questionnaire uses a 5-category Likert scale, where 0 equals ‘‘Strongly disagree’’ and 5 equals ‘‘Strongly agree’’.

Thirdly, the questionnaire “PSSUQ’’ was used to measure the good use of the simulator. This questionnaire has a Cronbach’s alpha coefficient and a range of 0.91 to 0.96, satisfactorily demonstrating its internal consistency and is composed of 19 items, using a 7-category Likert scale, where 1 is equivalent to “Strongly agree’’ and 7 to “Strongly disagree’’.

Exploratory Factor Analysis (EFA) following the Kaiser Criteria: Our EFA was carried out with a sample of 242 using principal component analysis with varimax rotation. We retained factors with eigenvalues greater than 1, following Kaiser’s criteria. The factor loadings were all above the 0.4 threshold, indicating a strong association of the items with the respective factors. This process helped to identify the underlying structure of the tool and to ensure that it measures distinct but related constructs.

The reliability of the questionnaires was assessed quantitatively using Cronbach’s alpha. The overall alpha coefficient for the tool indicated good internal consistency.

### Tools

The University Nursing Center Salus Infirmorum, attached to the University of Cádiz, created in 2009 the computer tool “Didactic simulator for learning nursing care’’. This simulator, after presenting the clinical case to the student, allows the student to make an assessment of the patient, following the model of Virginia Henderson, with the possibility of using tests or questionnaires that usually use the Diraya software, used in Hospitals and Health Centers of the Andalusian Health Service. The next step to be carried out by the student is to determine the diagnoses, objectives and interventions, also grouped in relation to the 14 basic needs of Virginia Henderson. The simulator informs the student whether the selected intervention is correct or not, and gives the score. After that, the student must complete the nursing records, divided into morning, afternoon and night shifts. The last item to be completed is the “nursing discharge’’.

### Statistical analysis

The statistical program SPSS 21 (Statistical Package for the Social Sciences) for Windows and the Office 2016 package were used to process the data.

The statistical analysis was carried out, firstly, by means of a descriptive analysis of the variables collected, calculating frequencies and percentages in the case of qualitative variables, and means and standard deviations, minimums and maximums for quantitative variables.

To analyze the relationships between variables, we first checked the normality of the quantitative variables by means of the Kolmogorov–Smirnov test, obtaining, after this analysis, that all the variables followed a non-normal distribution, and non-parametric tests were then carried out.

As for the analysis of the differences between the sociodemographic variables, comparisons of means were carried out using the Mann–Whitney U test for dichotomous variables, and the Kruskall-Wallis H test for variables with more than two categories.

Spearman correlation coefficients were calculated to analyze the relationship between age and scale.

In all analyses a significance level *p*-value < 0.05 was considered.

## Results

### Results of the “EHRNS” satisfaction scale

In order to know the level of Satisfaction that the student has after using the didactic Simulator in class, the EHRNS questionnaire has been used.

Next, the minimum and maximum values of the questionnaire, the mean and standard deviation will be shown, the most outstanding results will be commented and then the same will be done, but with the frequency and percentages of the tables.

When observing the comparison of the averages of student satisfaction with respect to the didactic simulator, it is worth mentioning that all the items are above 2.80 on a score in which “0” is the minimum value and “5” the maximum value. Except for the question “The assessment, diagnoses, objectives, interventions, nursing records and nursing discharge are subject to frequent failures in this program” with an average of 2.25, which, due to the type of question, when a low result is obtained, really means that the computer tool has few failures when it is used.

The question “Assessment, diagnoses, objectives, interventions, nursing records and nursing discharge are available in the computer program” is also significant data, with an average of 4.41 out of 5, so that the students were very satisfied with the availability in the computer program of the parts of the nursing process. The question “The program offers me enough resources to learn how to use the assessment, diagnoses, objectives, interventions, nursing records and nursing discharge” with an average of 4.12, so the students are also very satisfied with the resources of the software tool (Table 1).

**Table 1:** Means and standard deviations for nurse students level of satisfaction after using the didactic simulator in class

Ítem	Minimum	Maximum	Mean	Standard deviation
Assessment, diagnoses, goals, interventions, nursing records, and nursing discharge are available in the computer program.	0	5	4,41	0,866
Assessment, diagnoses, goals, interventions, nursing records, and nursing discharge are subject to frequent program failures	0	5	2,25	1,510
Assessment, diagnoses, goals, interventions, nursing records, and nursing discharge are easily managed in the program.	0	5	3,97	1,024
The program has sufficient support to manage assessment, diagnoses, goals, interventions, nursing records and nursing discharge.	0	5	4,05	0,943
The program provides me with sufficient resources to learn how to use the assessment, diagnoses, goals, interventions, nursing records and nursing discharge.	0	5	4,12	0,898
The data recorded regarding assessment, diagnoses, goals, interventions, nursing records, and nursing discharge are accurate and valid, according to the program.	0	5	3,99	0,859
Do you think students are concerned in the development of care plans?	0	5	3,70	1,163
Is it worth the time and effort required to use the program?	0	5	3,95	1,028
Overall, I am satisfied with the program’s assessment, diagnoses, goals, interventions, nursing records, and nursing discharge.	0	5	3,99	1,033
Overall, I am satisfied with the clinical use of the care plans in the program.	0	5	3,88	0,896
Assessment, diagnoses, goals, interventions, nursing records, and nursing discharge are done in an optimal time frame in the program.	0	5	3,71	1,041
Assessment, diagnoses, goals, interventions, nursing records, and nursing discharge in the program are appropriate.	0	5	3,98	0,935
The program facilitates effective communication between the majority of students and faculty regarding patient care plans.	0	5	3,92	0,932
The program plays a key role in the introduction of care plans in my facility.	0	5	3,83	0,957
The students should have had more say in the design of the program.	0	5	2,95	1,283
The program contributes to the safety of the students in the development of care plans.	0	5	3,84	0,957
The program contributes to the learning outcomes of students.	0	5	4,12	0,876
Program contributes to students’ knowledge of care plans.	0	5	3,95	0,945
Part of the cost increase in nurse education is due to computers.	0	5	2,85	1,291
I have first hand knowledge that problems with care plans have interfered with patient care.	0	5	3,10	1,370
The program would improve in the development of computerized care plans if the system could exchange information with other University Health Studies.	0	5	3,71	1,085

When analyzing the results of satisfaction by sub-scales, we highlight as significant data that the lowest average is 2.97 corresponding to the Quality of Information Logistics, out of a maximum score of 5, which is equivalent to strongly agree, so it is still a very good value. Thus, as an average of 3.99 as a maximum value in the Structural Quality (Table 2).

**Table 2:** Results of the subscales of satisfaction about the didactic simulator

Items	Minimum	Maximum	Mean	Standard deviation
Structural quality	0,00	5	3,99	0,719
Quality of information logistics	1,00	5	2,97	0,819
Effects quality results	0,00	5	3,81	0,805

Next, we will highlight the results of the most indicative frequencies and percentages according to the subscales of the questionnaire.

In order to evaluate the Structural Quality of the program, we observe that they present very favorable data, in this sense we can see how, for example, the item “The program contributes to the learning results of the students presents a 96.35 of satisfaction.

As significant data also the questions “The assessment, diagnoses, objectives, interventions, nursing records and nursing discharge are available in the computer program”, the students are satisfied either slightly, moderately or strongly with 96.2%, in addition, the question “The data recorded referring to the assessment, diagnoses, objectives, interventions, nursing records and nursing discharge are accurate and valid, according to the program” in which the students with 95% say they agree to a greater or lesser extent. “The program has sufficient support to manage assessment, diagnoses, objectives, interventions, nursing records and nursing discharge” the students responded with 94.6% that they agreed to some extent with the program support to manage clinical cases.

If we look at the Quality of Information, the question: “The program has a fundamental role in the introduction of care plans in my center” obtained a response rate of 91.3% positively, so we think that it would be a good tool for the student to use in the teaching–learning process of the Center.

If we analyze the data attending the subscale of the Quality of Results, the question “I have first-hand knowledge that, problems with care plans have interfered with patient care” the percentage is somewhat lower in this case, since only 71.8% of the students agreed with this item.

Finally, the question “The program would improve in the elaboration of computerized care plans if the system could exchange information with other University Health Studies”, the students agreed with 88.4% (Table 3).

**Table 3:** Results of frequencies and percentages of the satisfaction scale on the didactic simulator

Ítems	Strongly disagree	Moderately disagree	Slightly disagree	Slightly agree	Moderately agree	Strongly agree
Assessment, diagnoses, goals, interventions, nursing records, and nursing discharge are available in the computer program.	1 (0,4%)	1 (0,4%)	7 (2,9%)	24 (9,9%)	64 (26,4%)	145 (59,9%)
Assessment, diagnoses, goals, interventions, nursing records, and nursing discharge are subject to frequent program failures	40 (16,6%)	43 (17,8%)	46 (19,1%)	58 (24,1%)	37 (15,4%)	17 (7,1%)
Assessment, diagnoses, goals, interventions, nursing records, and nursing discharge are easily managed in the program.	1 (0,4%)	2 (0,8%)	21 (8,7%)	45 (18,6%)	83 (34,3%)	90 (37,2%)
The program has sufficient support to manage assessment, diagnoses, goals, interventions, nursing records and nursing discharge.	1 (0,4%)	3 (1,2%)	9 (3,7%)	47 (19,4%)	93 (38,4%)	89 (36,8%)
The program provides me with sufficient resources to learn how to use the assessment, diagnoses, goals, interventions, nursing records and nursing discharge.	1 (0,4%)	4 (1,7%)	9 (3,7%)	23 (9,5%)	118 (48,8%)	87 (36,0%)
The data recorded regarding assessment, diagnoses, goals, interventions, nursing records, and nursing discharge are accurate and valid, according to the program.	1 (0,4%)	2 (0,8%)	9 (3,7%)	41 (16,9%)	122 (50,4%)	67 (27,7%)
Do you think students are concerned in the development of care plans?	3 (1,2%)	10 (4,1%)	21 (8,7%)	56 (23,2%)	83 (34,4%)	68 (28,2%)
Is it worth the time and effort required to use the program?	1 (0,4%)	8 (3,3%)	9 (3,8%)	47 (19,6%)	93 (38,8%)	82 (34,2%)
Overall, I am satisfied with the program’s assessment, diagnoses, goals, interventions, nursing records, and nursing discharge.	5 (2,1%)		13 (5,4%)	40 (16,6%)	99 (41,1%)	84 (34,9%)
Overall, I am satisfied with the clinical use of the care plans in the program.	1 (0,4%)	2 (0,8%)	12 (5,0%)	55 (22,7%)	112 (46,3%)	60 (24,8%)
Assessment, diagnoses, goals, interventions, nursing records, and nursing discharge are done in an optimal time frame in the program.	4 (1,7%)	4 (1,7%)	16 (6,6%)	63 (26,0%)	101 (41,7%)	54 (22,3%)
Assessment, diagnoses, goals, interventions, nursing records, and nursing discharge in the program are appropriate.	1 (0,4%)	2 (0,8%)	15 (6,2%)	40 (16,6%)	107 (44,4%)	76 (31,5%)
The program facilitates effective communication between the majority of students and faculty regarding patient care plans.	1 (0,4%)	2 (0,8%)	12 (5,0%)	57 (23,6%)	98 (40,5%)	72 (29,8%)
The program plays a key role in the introduction of care plans in my facility.	1 (0,4%)	3 (1,2%)	17 (7,0%)	56 (23,1%)	104 (43,0%)	61 (25,2%)
The students should have had more say in the design of the program.	8 (3,3%)	24 (10,0%)	51 (21,3%)	78 (32,5%)	47 (19,6%)	32 (13,3%)
The program contributes to the safety of the students in the development of care plans.	1 (0,4%)	3 (1,2%)	12 (5,0%)	68 (28,2%)	90 (37,3%)	67 (27,8%)
The program contributes to the learning outcomes of students.	1 (0,4%)	1 (0,4%)	7 (2,9%)	43 (17,8%)	98 (40,5%)	92 (38,0%)
Program contributes to students’ knowledge of care plans.	2 (0,8%)	4 (1,7%)	8 (3,3%)	46 (19,1%)	112 (46,5%)	69 (28,6%)
Part of the cost increase in nurse education is due to computers.	13 (5,4%)	24 (10,0%)	47 (19,5%)	81 (33,6%)	53 (22,0%)	23 (9,5%)
I have first hand knowledge that problems with care plans have interfered with patient care.	15 (6,2%)	17 (7,1%)	36 (14,9%)	70 (29,0%)	66 (27,4%)	37 (15,4%)
The program would improve in the development of computerized care plans if the system could exchange information with other University Health Studies.	3 (1,3%)	7 (2,9%)	18 (7,5%)	60 (25,0%)	93 (38,8%)	59 (24,6%)

### Results of the “PSSUQ” usage scale

The purpose of the PSSUQ questionnaire is to measure the good use of the didactic simulator, the quality of the information and the quality of the interface. It will allow us to know the level of use of our tool by the student.

In the results of the use of the computer tool by the students, we highlight as significant data that all the items present an average equal to or lower than 3.04 out of 7, where “1” corresponds to a lot of use and “7” to little use.

On the other hand, we observe that the lowest score corresponds to the question “In general I am satisfied with this program” with an average of 2.20. Therefore, the students in general are quite satisfied with this program and the highest score refers to the item “Error messages appeared in the program that helped me later to solve the problems” with an average of 3.04. This can be interpreted as meaning that a percentage of students, close to half of those surveyed, consider that the error messages do not help them to subsequently solve the problems they encounter when they are using the didactic simulator (Table 4).

**Table 4:** Results of means and standard deviations of the didactic simulator usage scale

Items	Minimum	Maximum	Mean	Standard deviation
Overall, I am satisfied with how easy it was to use this Health program.	1	7	2,41	1,440
It was simple for me to use this software.	1	7	2,52	1,519
I was able to easily complete clinical cases using this program	1	7	2,48	1,406
I was able to quickly complete clinical cases using this program	1	7	2,88	1,549
I was able to efficiently complete clinical cases using this program	1	7	2,77	1,531
I felt comfortable using this program	1	7	2,62	1,561
I found it easy to learn how to use this program	1	7	2,43	1,454
I think I could become a better learner using this program	1	7	2,69	1,480
Error messages appeared in the program that subsequently helped me to troubleshoot problems	1	7	3,04	1,837
Whenever I made a mistake using the program I could solve it easily and quickly.	1	7	2,81	1,589
The information provided by the program was clear; e.g. online help, on-screen messages, etc.	1	7	2,57	1,512
It was easy for me to find the information I needed in the program.	1	7	2,61	1,44
The information provided by the program was easy to understand.	1	7	2,45	1,474
The information was effective in helping me complete the cases.	1	7	2,38	1,40
The organization of the information on the program screens is clear.	1	7	2,35	1,374
The system interface is pleasant	1	7	2,58	1,501
I enjoyed using the interface of this system	1	7	2,61	1,496
This program has all the features I expected it to have	1	7	2,46	1,458
Overall I am satisfied with this program	1	7	2,20	1,414

When analyzing the data by subscales, we see that the averages are between 2.56 and 2.59 out of a maximum of 6.29. This means, as in the previous scale, knowing that “1” corresponds to much use, that in general the data on the use of the program are optimal (Table 5).

**Table 5:** Results of the didactic simulator use subscales

Items	Minimum	Maximum	Mean	Standard deviation
System usage	1	6,29	2,59	1,313
Program quality	1	6,08	2,56	1,246

## Discussion

Healthcare ICTs have been mainly studied in medical areas. There are few studies that analyze this type of technology in the fields dedicated to nursing [31–33], so our intervention is opening the field of knowledge in nursing science.

The use of simulators in nursing is a widespread practice for the improvement of student learning as reported in the literature review by Nsouli and Vlachopoulos [34], which indicates that this type of technology has more use than management and continuity of care programs. The didactic simulator provided in our study is the first of those currently available that performs the nursing care process in a comprehensive manner. Since the NNN consult does not have access to the questionnaires and scales that nurses can assess for their patients’ altered human responses and subsequent nursing discharge [22]. In addition, nursing-focused studies conduct most of their research in hospital-based problem-solving programs [35]. However, the simulation tool of the present research is valid for the entire care area, whether in the hospital setting or in health centers.

### Satisfaction with the program

Today’s healthcare environment is dynamic and rapidly changing in line with modern technological advances. To keep pace with these changes, the nursing profession must respond and integrate appropriate technologies, especially in educational settings [36]. Due to the COVID-19 pandemic, the importance of being able to further enhance nursing knowledge through simulators and technologies that could perform their work virtually was objectified and although distance learning with ICT support was considered moderately effective by students [37]. Arunasalam noted that nursing students experience some frustration with the use of ICTs [38]. However, satisfaction with the simulation tool by students has been adequate in our study, a fact that we could observe in research from Austria indicating that Filipino nursing students had a positive attitude toward technology in nursing education [39]. The high scores on the structural quality of the program in our study were enhanced by the aforementioned student satisfaction. This fact refers us to the literature review by Fagerström et al. [40] which informs us that the majority of nurses think that ICTs improve the quality of health system services even though there is a minority sector that thinks otherwise.

### Use of the program

The pace of the creation of programs, platforms and simulators in nursing practice has been slow [40]. However, once these tools have been created, we can observe the good use reported by the participants. On the other hand, current systematic reviews related to healthcare technologies report that more research is still needed on the topic [35].

We currently find that, compared with other health care professionals, nurses appear to be less likely to use computers. However, the students participating in our study have reported that the use of the simulation program is clear, easy to understand and helps them with their daily work. This fact can also be observed in the healthcare environment where there is a favorable integration and good use of ICTs even though nurses report that they do not feel prepared for it [40]. However, Parada et al. report that despite the feeling of lack of preparedness, nurses themselves have started to make use of such programmes in order to ensure proper care, coverage and continuity of care, improve communication processes and match the available health resources to the high demands [41].

The results reported in the review by Fagerström et al. [40] on the ease of use of this type of program indicate that it can be a source of stress reduction for students and in the health care setting, a fact that leads us to one of the benefits that our simulator could have through its continued use.

In relation to the error messages reported by the program, the students have observed that it does not help them to solve them. This also occurs in the research of Johansson et al. [42] who refer us that functional problems with certain programs negatively affect the use of the program by nurses.

Krick et al. in their review informs us that there are not enough quality studies to compare the effects of certain ICTs such as simulators. This is because most innovative technologies are under development or have never reached the implementation phase [35]. However, this is not the case with our simulator, which has been implemented in the university environment.

Pugoy et al. [43] Austria [39] and Peñaflor-Espinosa [44] reported that nursing education using ICTs was more effective than traditional methods. However, the Souza-Junior study reports that there are no significant differences between the two methods [45], but these types of services have been found to work better when used in line with traditional methods [46].

To conclude, Camejo Giménez [47] in his research reports that teachers recognise the importance of education with computerised and virtual learning programmes in Nursing in order to create academic processes and work procedures that aim at the permanent expression of educational action levels that are increasingly satisfactory and adapted to global development.

This tool should be implemented in the nursing degree with the objective of learning through a format similar to that used in the national health system to make nursing care plans, with their respective follow-ups and assessments through questionnaires and finally hospital discharge.

Thanks to this, the students would achieve the correct use of health ICTs and the implementation of nursing knowledge in their daily work life in a satisfactory way.

The limited bibliography on the subject leads us to consider that it would be advisable to continue implementing the tool in other nursing universities and that the data could be compared in different geographical areas. Furthermore, it would be positive to constantly update the nursing diagnoses, objectives and interventions according to the latest NANDA consensus.

## Strengths and limitations

The strengths of the present study are related to the lack of programmes that relate all the variables presented in a single simulation application, as there are no discharge management programmes that contain nursing assessment systems or vice versa. However, our tool achieves the correct synergy of these two aforementioned variables. On the other hand, one of the limitations of the study is related to the transversality of the study, since it is not possible to make a complete follow-up over time of the use of the tool.

## Conclusions

The implication of ICT in the teaching–learning process among Nursing Degree students, it is considered optimal.

Regarding the opinion on the Satisfaction of the computer tool created by us for the learning of the Nursing Process in Nursing degree students, in all the subscales of the Satisfaction questionnaire, it is detected that the students value very positively the didactic Simulator, specifically the dimension of the quality of the information reaches the highest level.

Older students show a lower level of satisfaction with the items of the program compared to younger students. Likewise, when we refer to it as an aid for effective communication between teachers and students in relation to the Care Plans, the oldest subjects are the least satisfied.

Considering the opinion of the use of the didactic simulator used, we can conclude respect to the subscales of the Use of the program, the students rated all of them positively, obtaining in this case a higher score for the quality of the interface.

Older students have a lower use of personal interaction programs, as well as a higher use of multimedia devices, compared to younger students.

## Data Availability

Not applicable.

## Abbreviations

ICT: Information and Communication Technology
DISILNP: Didactic Simulator for Learning the Nursing Process
NCP: Nursing Care Process
NANDA: North American Nursing Diagnosis Association
NOC: Nursing Outcome Classification
NIC: Nursing Interventions Classification
EHRNS: Electronic Health Record Nurse Satisfaction
PSSUQ: Post Study System Usability Questionnaire
SPSS: Statistical Package for the Social Sciences
